# Supplementary material for: Accounting for heterogeneity due to environmental sources in meta-analysis of genome-wide association studies
Source: Commun Biol. 2024 Nov 14;7:1512. doi: 10.1038/s42003-024-07236-9 (PMC11564974; doi:10.1038/s42003-024-07236-9)

Supplementary Information

Accounting for heterogeneity due to environmental sources in meta-analysis of genome-wide association studies

Siru Wang[#], Oyesola O. Ojewunmi[#], Abram Kamiza, Michele Ramsay, Andrew P Morris, Tinashe Chikowore[##], Segun Fatumo[##], Jennifer L Asimit[##]

[#] Equal contributing first authors
[##] Equal contributing last authors

Supplementary Table 1. Heterogeneity scenarios parameterised in terms of β in each reference sub-population. These allelic effect patterns are used in simulation 1 for gene-sex interactions and simulation 2 for gene-smoker interactions, where both genders have the same model but differ in smoking proportions within each of the eight populations. From Phase 3 of the 1000 Genomes project, we include West-central Africa (ESN,YRI), West Africa (GWD,MSL), and one population of East Africa (LWK), and we also include two Zulu cohorts from Southern Africa (DCC, DDS) and the Uganda Genome Resource from East Africa (UGD).

Population		Heterogeneity scenarios (allelic effect β)					
Code	Region	Ancestrally Homogeneous	East Africa	West-central Africa	West Africa	South Africa	Non-ancestral Africa
ESN	West-central Africa	β	0	β	0	0	β
YRI	West-central Africa	β	0	β	0	0	0
GWD	West Africa	β	0	0	β	0	β
MSL	West Africa	β	0	0	β	0	0
DCC	South Africa	β	0	0	0	β	β
DDS	South Africa	β	0	0	0	β	0
LWK	East Africa	β	β	0	0	0	β
UGD	East Africa	β	β	0	0	0	0

Supplementary Table 2. False positive error rates (type 1 errors), at a nominal significance threshold (P < 0.05) to detect association from env-MR-MEGA and MR-MEGA across a range of heterogeneity scenarios are well-calibrated. The type I errors were estimated as the probability that the causal variant has P-value of association less than 0.05 based on 1000 replications.

Heterogeneity scenario	Unequal sample size		Equal sample size	
	env-MR-MEGA	MR-MEGA	env-MR-MEGA	MR-MEGA
Ancestrally homogeneous	0.041	0.046	0.038	0.031
East Africa	0.041	0.046	0.038	0.031
West-central Africa	0.041	0.046	0.038	0.031
West Africa	0.041	0.046	0.038	0.031
South Africa	0.041	0.046	0.038	0.031
Non-ancestral Africa	0.041	0.046	0.038	0.031

Supplementary Table 3. **In the homogeneity in sex and ancestry scenario all tests for heterogeneity in allelic effects due to environment and/or ancestry are well-calibrated.** Here, male and female cohorts share the same allelic effects**.** Power to detect heterogeneity due to ancestry and/or environment were assessed at $P < 0.05$. "AEhet" refers to the power to detect heterogeneity due to ancestry and environment, "Ahet" refers to the power to detect heterogeneity due to ancestry alone and "Ehet" refers to the power to detect heterogeneity due to environment alone.

env-MR-MEGA: unequal sample size			
β	AEhet	Ahet	Ehet
0	0.048	0.054	0.045
0.02	0.048	0.054	0.045
0.04	0.048	0.054	0.045
0.06	0.048	0.054	0.045
0.08	0.048	0.054	0.045
0.1	0.048	0.054	0.045
env-MR-MEGA: equal sample size			
0	0.049	0.040	0.053
0.02	0.049	0.040	0.053
0.04	0.049	0.040	0.053
0.06	0.049	0.040	0.053
0.08	0.049	0.040	0.053
0.1	0.049	0.040	0.053

Supplementary Table 4. Smoker proportion settings for the setting where female and male cohorts have similar smoker rates. The eight populations from different African regions were stratified by sex.

Populations		Smoker proportions (female/male)		
Code	Region	Non-difference	Same direction	Mixed direction
DCC	South Africa	0.149/0.149	0.088/0.226	0.088/0.226
DDS	South Africa	0.644/0.644	0.617/0.719	0.719/0.617
UGD	East Africa	0.659/0.659	0.593/0.691	0.691/0.593
LWK	East Africa	0.135/0.135	0.12/0.231	0.12/0.231
ESN	West-central Africa	0.655/0.655	0.604/0.714	0.714/0.604
YRI	West-central Africa	0.163/0.163	0.096/0.202	0.096/0.202
GWD	West Africa	0.676/0.676	0.595/0.749	0.749/0.595
MSL	West Africa	0.15/0.15	0.075/0.235	0.075/0.235

Supplementary Table 5. Smoker proportion settings for the setting where female and male cohorts have very different smoker rates. The eight populations from different African regions were stratified by sex.

Populations		Smoker proportions (female/male)	
Code	Region	Same direction (lower proportion in female)	Mixed direction
DCC	South Africa	0.128/0.631	0.631/0.128
DDS	South Africa	0.152/0.652	0.152/0.652
UGD	East Africa	0.165/0.876	0.876/0.165
LWK	East Africa	0.243/0.81	0.81/0.243
ESN	West-central Africa	0.215/0.836	0.215/0.836
YRI	West-central Africa	0.061/0.888	0.888/0.061
GWD	West Africa	0.192/0.676	0.192/0.676
MSL	West Africa	0.066/0.655	0.066/0.655

Supplementary Figure 1. Axes of genetic variation separating eight African ancestry populations. The first two axes of genetic variation from multi-dimensional scaling of the Euclidean distance matrix between 8 populations are sufficient to separate population groups from different regions of Africa: East Africa (LWK, UGD), South Africa (DCC,DDS), West-central Africa (ESN, YRI) and West Africa (GWD, MSL).

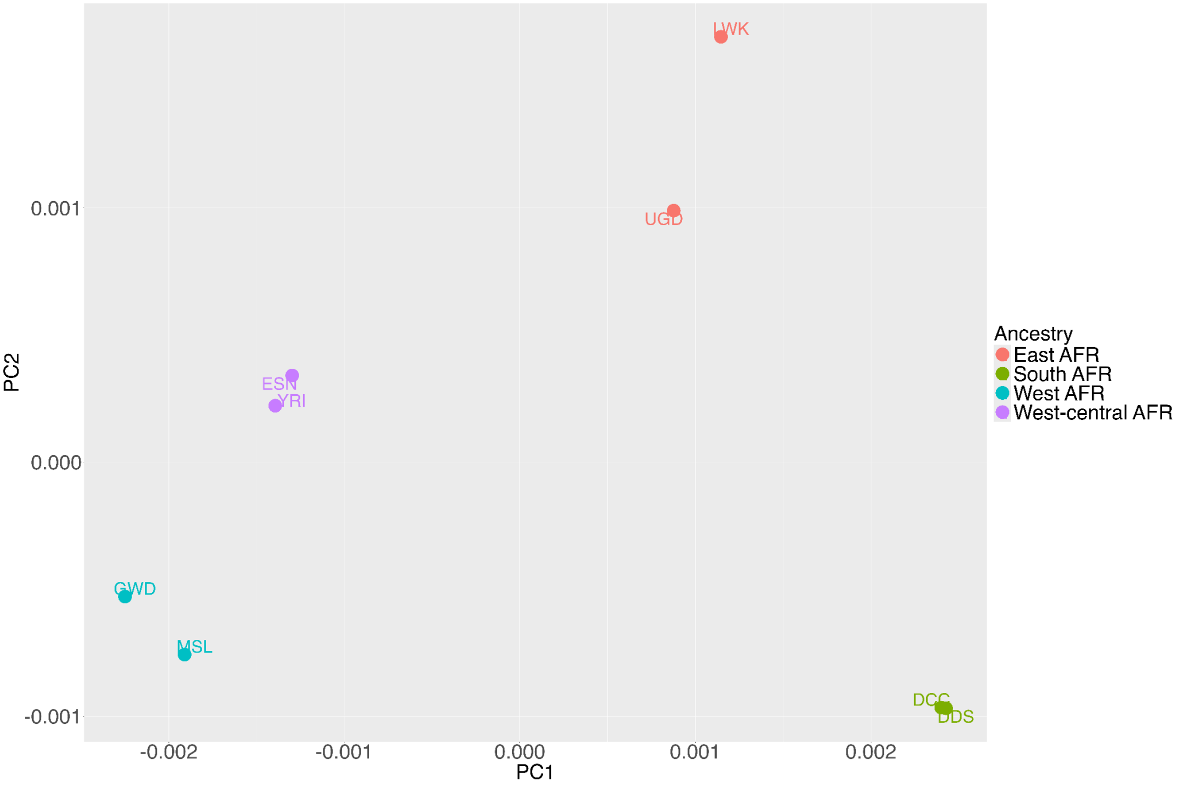

Supplementary Figure 2. Across six heterogeneity scenarios, where the 16 sex-stratified cohorts have unequal sample sizes (≥3000 in each female/male cohort), power for heterogeneity of allelic effects due to ancestry and environment from env-MR-MEGA was greater than power for heterogeneity of allelic effects due to ancestry alone from MR-MEGA. “env_MR_MEGA_AEhet” (red line) corresponds to the power to detect heterogeneity due to ancestry and environment attained from env-MR-MEGA; “env_MR_MEGA_Ahet” (green line) corresponds to the power to detect heterogeneity due to ancestry alone attained from env-MR-MEGA; “env_MR_MEGA_Ehet” (blue line) corresponds to the power to detect heterogeneity due to environment alone attained from env-MR-MEGA; “MR_MEGA_Ahet” (purple line) corresponds to the power to detect heterogeneity due to ancestry obtained from MR-MEGA. Power to detect heterogeneity due to ancestry and/or environment was assessed at $P < 0.05$ and based on 1000 replications.

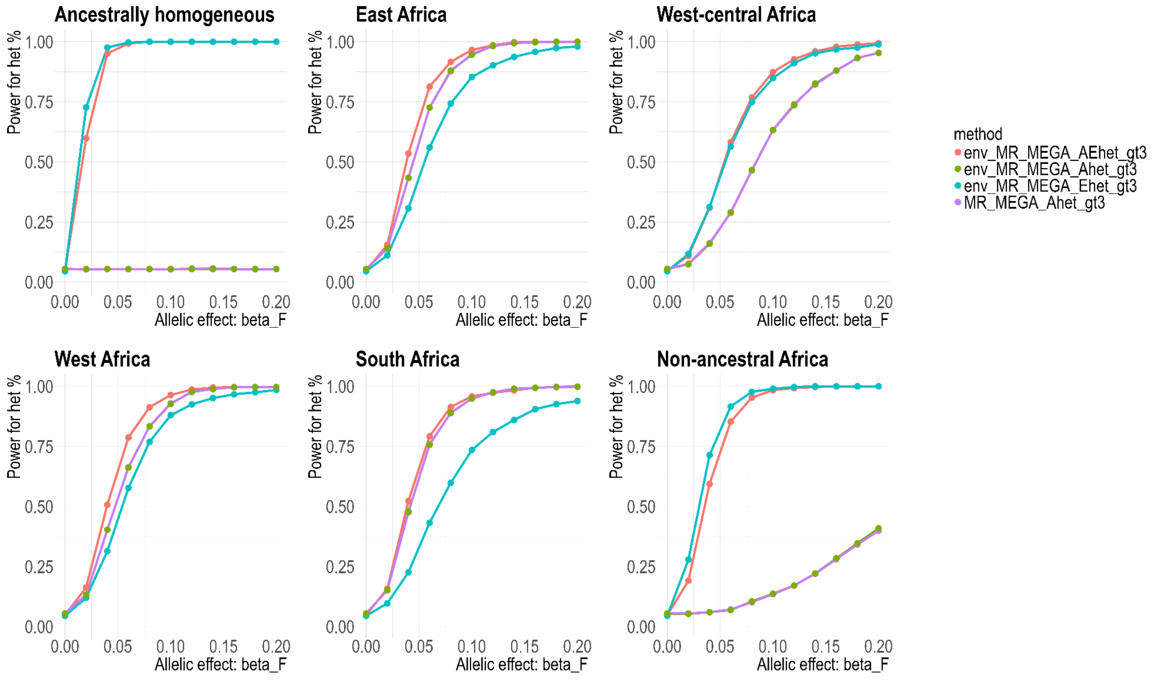

Supplementary Figure 3. Across six heterogeneity scenarios where the 16 sex-stratified cohorts share equal sample sizes (1000 in each female/male cohort), power for heterogeneity of allelic effects due to ancestry and environment from env-MR-MEGA was greater than power for heterogeneity of allelic effects due to ancestry and environment from MR-MEGA. "env_MR_MEGA_AEhet" (red line) corresponds to the power to detect heterogeneity due to ancestry and environment attained from env-MR-MEGA; "env_MR_MEGA_Ahet" (green line) corresponds to the power to detect heterogeneity due to ancestry alone only attained from env-MR-MEGA; "env_MR_MEGA_Ehet" (blue line) corresponds to the power to detect heterogeneity due to environment alone attained from env-MR-MEGA; "MR_MEGA_Ahet" (purple line) corresponds to the power to detect heterogeneity due to ancestry attained from MR-MEGA. Power to detect heterogeneity due to ancestry and/or environment was assessed at $P < 0.05$ and based on 1000 replications.

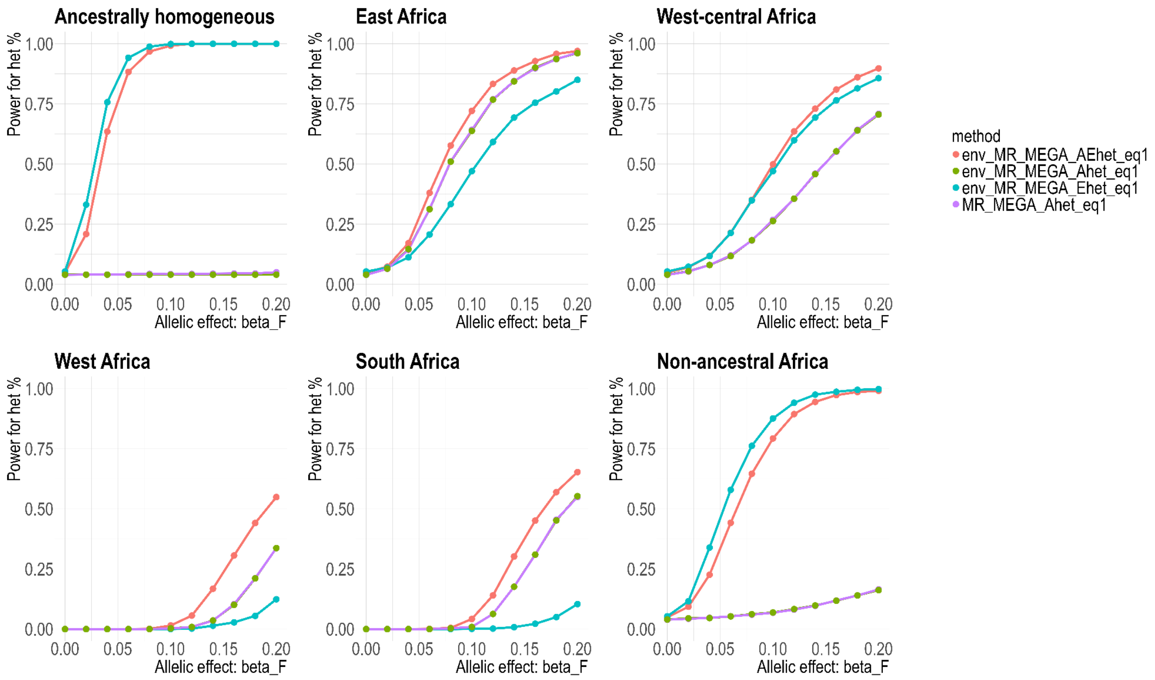

Supplementary Figure 4. Across six heterogeneity scenarios involving 16 sex-stratified cohorts, where female and male cohorts in the same population share the same smoking proportion, env-MR-MEGA exhibits higher power in detecting association compared to MR-MEGA, especially in ancestrally homogeneous, west-central Africa and non-ancestral Africa scenarios. "env_MR_MEGA_assoc" and "MR_MEGA_assoc" refer to the power to detect association obtained from env-MR-MEGA and MR-MEGA. Power was assessed at $P < 5 \times 10^{-8}$ and based on 1000 replications with unequal sample sizes (≥3000 in each cohort).

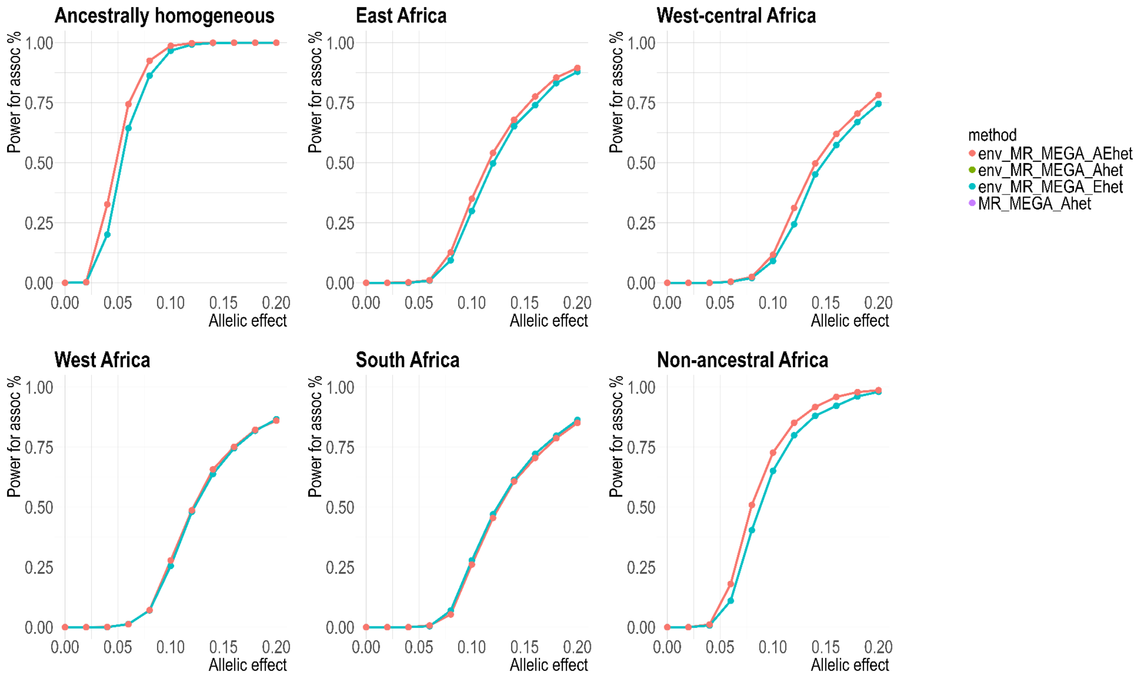

Supplementary Figure 5. Across six heterogeneity scenarios involving 16 sex-stratified cohorts, where minor reductions in smoking proportions between male and female cohorts occur (same direction), env-MR-MEGA exhibits higher power in detecting association compared to MR-MEGA, especially in ancestrally homogeneous, west-central Africa and non-ancestral Africa scenarios. "env_MR_MEGA_assoc" and "MR_MEGA_assoc" refer to the power to detect association obtained from env-MR-MEGA and MR-MEGA. Power was assessed at $P < 5 \times 10^{-8}$ and based on 1000 replications with unequal sample sizes (≥3000 in each cohort).

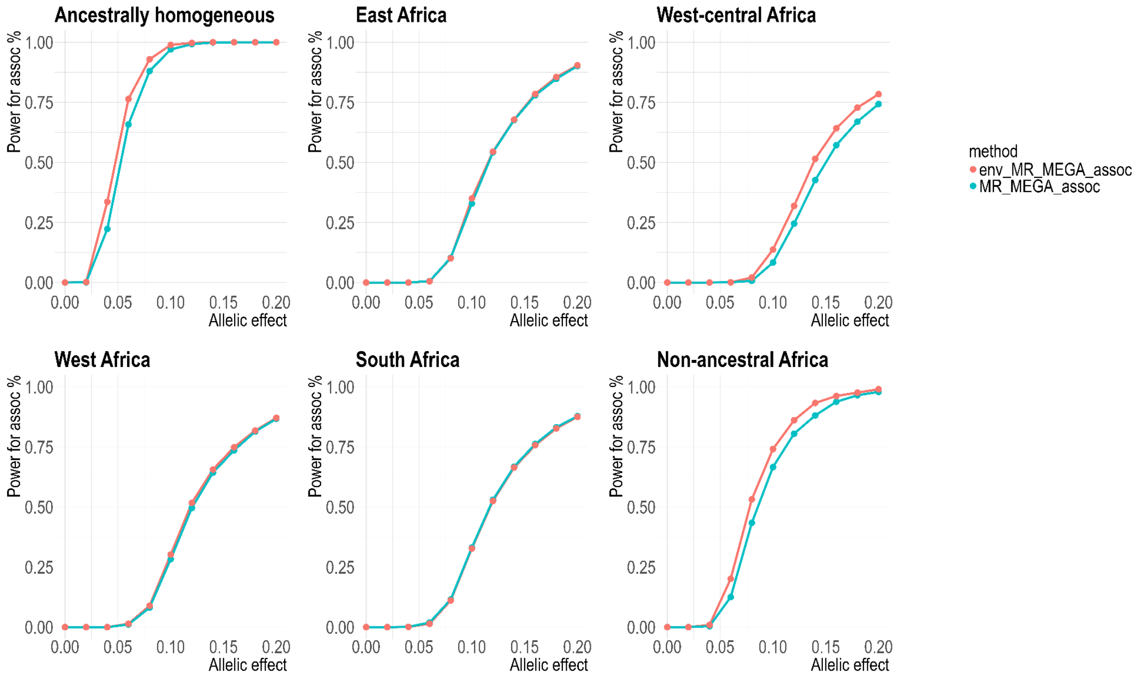

Supplementary Figure 6. When female and male cohorts from the same population share the same smoking proportion across six heterogeneity scenarios involving 16 sex-stratified cohorts, power for heterogeneity due to ancestry and environment from env-MR-MEGA exceeded power for heterogeneity due to ancestry from MR-MEGA, particularly in ancestral homogeneity, west-central Africa and non-ancestral Africa scenarios. "env_MR_MEGA_AEhet" (red line) corresponds to the power to detect heterogeneity due to ancestry and environment attained from env-MR-MEGA; "env_MR_MEGA_Ahet" (green line) corresponds to the power to detect heterogeneity due to ancestry alone attained from env-MR-MEGA; "env_MR_MEGA_Ehet" (blue line) corresponds to the power to detect heterogeneity due to environment alone attained from env-MR-MEGA; "MR_MEGA_Ahet" (purple line) corresponds to the power to detect heterogeneity due to ancestry attained from MR-MEGA. Power to detect heterogeneity due to ancestry and/or environment was assessed at $P < 0.05$ and based on 1000 replications with unequal sample sizes (≥3000 in each cohort).

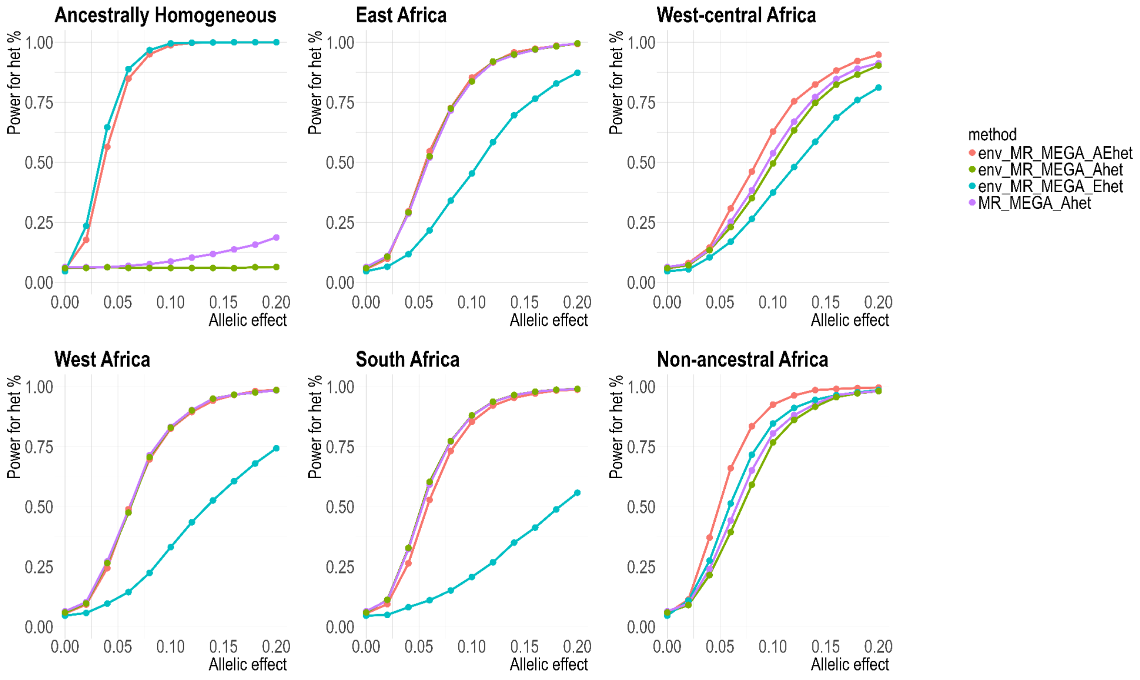

Supplementary Figure 7. When minor random reductions or increases in smoking proportions between male and female cohorts occur (mixed direction) across six heterogeneity scenarios involving 16 sex-stratified cohorts, power for heterogeneity in allelic and environmental effects from env-MR-MEGA exceeded power for heterogeneity in allelic effects from MR-MEGA, particularly in ancestral homogeneity, west-central Africa and non-ancestral Africa scenarios. "env_MR_MEGA_AEhet" (red line) corresponds to the power to detect heterogeneity due to ancestry and environment attained from env-MR-MEGA; "env_MR_MEGA_Ahet" (green line) corresponds to the power to detect heterogeneity due to ancestry alone attained from env-MR-MEGA; "env_MR_MEGA_Ehet" (blue line) corresponds to the power to detect heterogeneity due to environment alone attained from env-MR-MEGA; "MR_MEGA_Ahet" (purple line) corresponds to the power to detect heterogeneity due to ancestry attained from MR-MEGA. Power to detect heterogeneity due to ancestry and/or environment was assessed at $P < 0.05$ and based on 1000 replications with unequal sample sizes (≥3000 in each cohort).

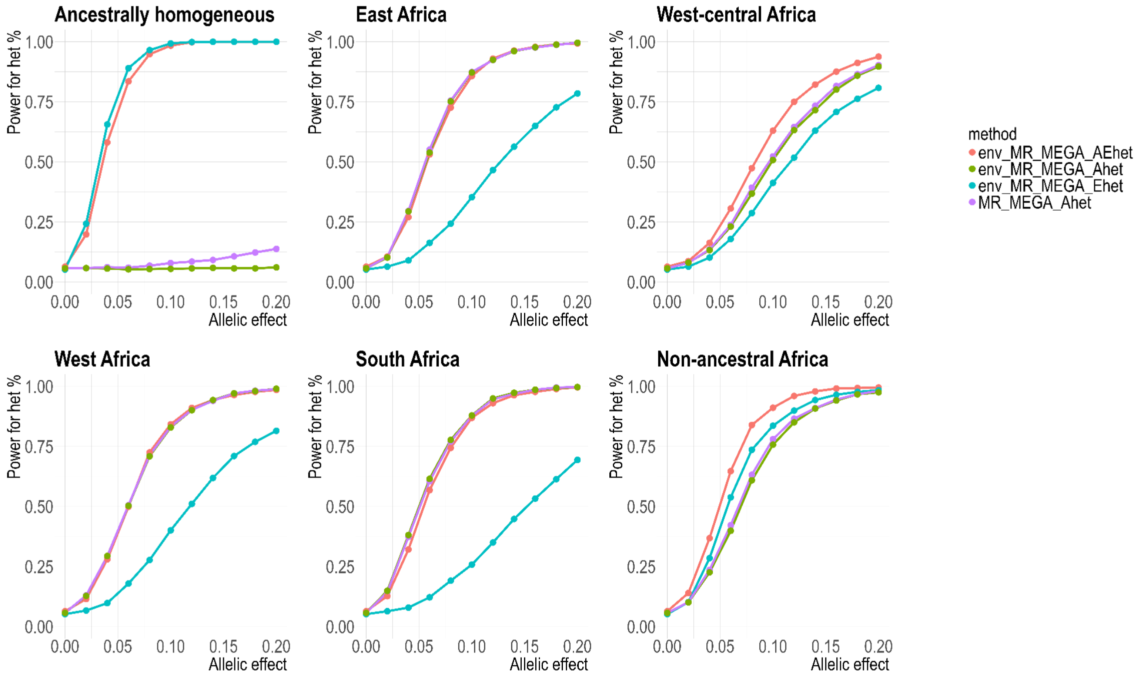

Supplementary Figure 8. When minor reductions in smoking proportions in female cohorts occur (same direction) across six heterogeneity scenarios involving 16 sex-stratified cohorts, power for heterogeneity due to ancestry and environment from env-MR-MEGA exceeded power for heterogeneity due to ancestry from MR-MEGA, particularly in ancestral homogeneity, west-central Africa and non-ancestral Africa scenarios. "env_MR_MEGA_AEhet" (red line) corresponds to the power to detect heterogeneity due to ancestry and environment attained from env-MR-MEGA; "env_MR_MEGA_Ahet" (green line) corresponds to the power to detect heterogeneity due to ancestry alone attained from env-MR-MEGA; "env_MR_MEGA_Ehet" (blue line) corresponds to the power to detect heterogeneity due to environment alone attained from env-MR-MEGA; "MR_MEGA_Ahet" (purple line) corresponds to the power to detect heterogeneity due to ancestry attained from MR-MEGA. Power to detect heterogeneity due to ancestry and environment was assessed at $P < 0.05$ and based on 1000 replications with unequal sample sizes (≥3000 in each cohort).

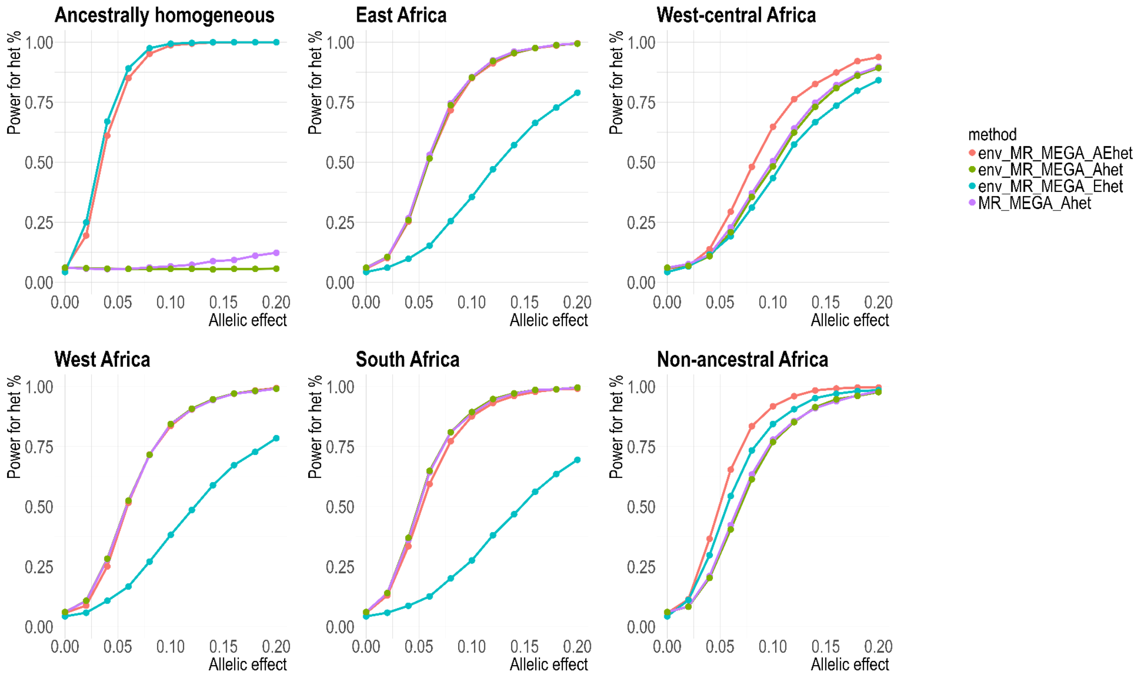

Supplementary Figure 9. In the South Africa heterogeneity scenario, where allelic heterogeneity is more strongly correlated with ancestry compared to varied smoking proportions across 12 sex-stratified cohorts (no-difference, mixed direction and same direction), the power for allelic heterogeneity due to ancestry attained from both env-MR-MEGA and MR-MEGA is moderately greater than that due to ancestry and environment for moderate allelic effects. The power is measured as the proportion of replications that detect the associated variants. The error bar shows 95% confidence levels, defined as power $\pm$ SEM, where SEM is the standard proportion error bound of a 95% confidence interval. “env_MR_MEGA_AEhet” (red line) corresponds to the power to detect heterogeneity due to ancestry and environment attained from env-MR-MEGA; “env_MR_MEGA_Ahet” (green line) corresponds to the power to detect heterogeneity due to ancestry alone attained from env-MR-MEGA; “MR_MEGA_Ahet” (blue line) corresponds to the power to detect heterogeneity due to ancestry attained from MR-MEGA. Power was assessed at $P < 0.05$ and based on 1000 replications with unequal sample sizes (≥3000 in each cohort).

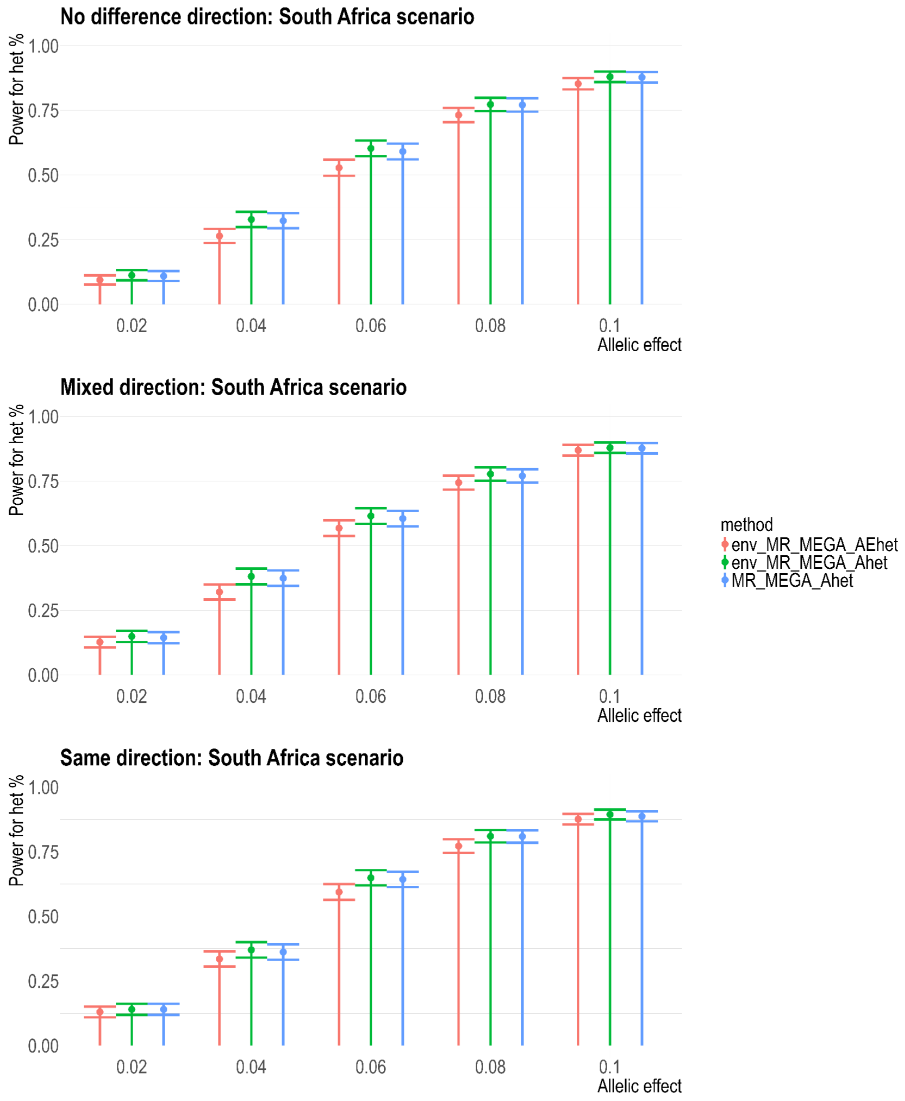

Supplementary Figure 10. When notable random reductions or increases in smoking proportions between male and female cohorts occur (mixed direction) across six heterogeneity scenarios involving 16 sex-stratified cohorts, power for heterogeneity due to ancestry and environment from env-MR-MEGA exceeded power for heterogeneity due to ancestry from MR-MEGA, particularly in ancestrally homogeneity, west-central Africa and non-ancestral Africa scenarios. "env_MR_MEGA_AEhet" (red line) corresponds to the power to detect heterogeneity due to ancestry and environment attained from env-MR-MEGA; "env_MR_MEGA_Ahet" (green line) corresponds to the power to detect heterogeneity due to ancestry alone attained from env-MR-MEGA; "env_MR_MEGA_Ehet" (blue line) corresponds to the power to detect heterogeneity due to environment alone attained from env-MR-MEGA; "MR_MEGA_Ahet" (purple line) corresponds to the power to detect heterogeneity due to ancestry alone attained from MR-MEGA. Power to detect heterogeneity due to ancestry and/or environment was assessed at $P < 0.05$ and based on 1000 replications with unequal sample sizes (≥3000 in each cohort).

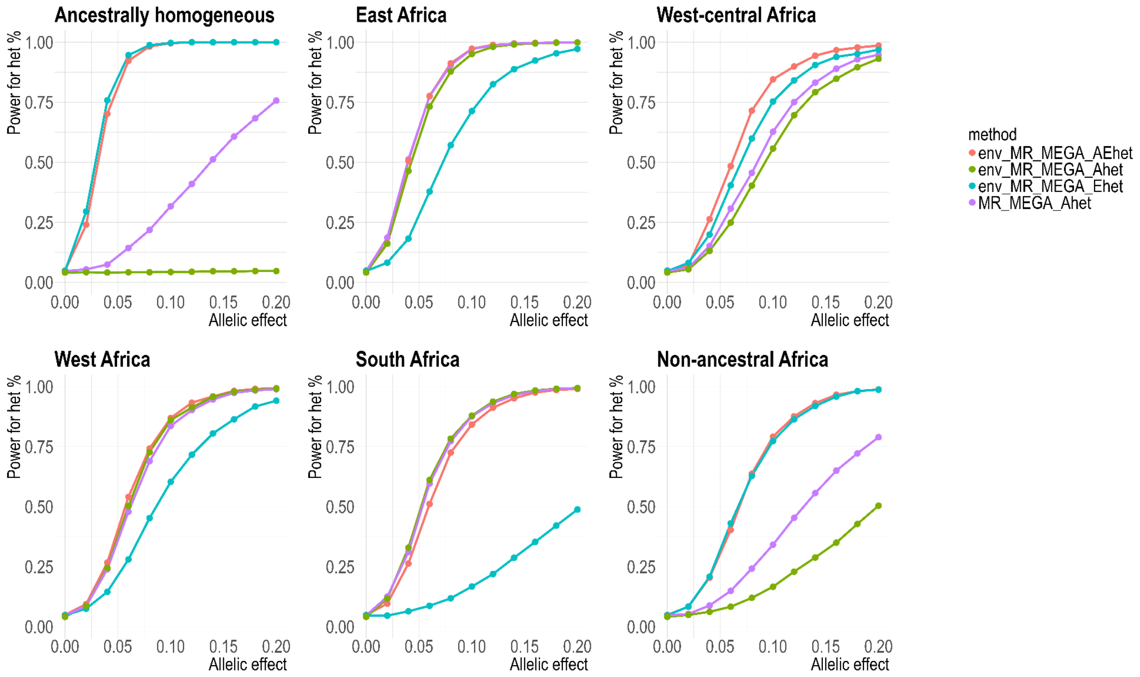

Supplementary Figure 11. When notable reductions in smoking proportions in female cohorts occur (same direction) across six heterogeneity scenarios involving 16 sex-stratified cohorts, power for heterogeneity due to ancestry and environment from env-MR-MEGA exceeded power for heterogeneity due to ancestry from MR-MEGA, particularly in ancestrally homogeneity, west-central Africa and non-ancestral Africa scenarios. "env_MR_MEGA_AEhet" (red line) corresponds to the power to detect heterogeneity due to ancestry and environment attained from env-MR-MEGA; "env_MR_MEGA_Ahet" (green line) corresponds to the power to detect heterogeneity due to ancestry alone attained from env-MR-MEGA; "env_MR_MEGA_Ehet" (blue line) corresponds to the power to detect heterogeneity due to environment alone attained from env-MR-MEGA; "MR_MEGA_Ahet" (purple line) corresponds to the power to detect heterogeneity due to ancestry attained from MR-MEGA. Power to detect heterogeneity due to ancestry and/or environment was assessed at $P < 0.05$ and based on 1000 replications with unequal sample sizes (≥3000 in each cohort).

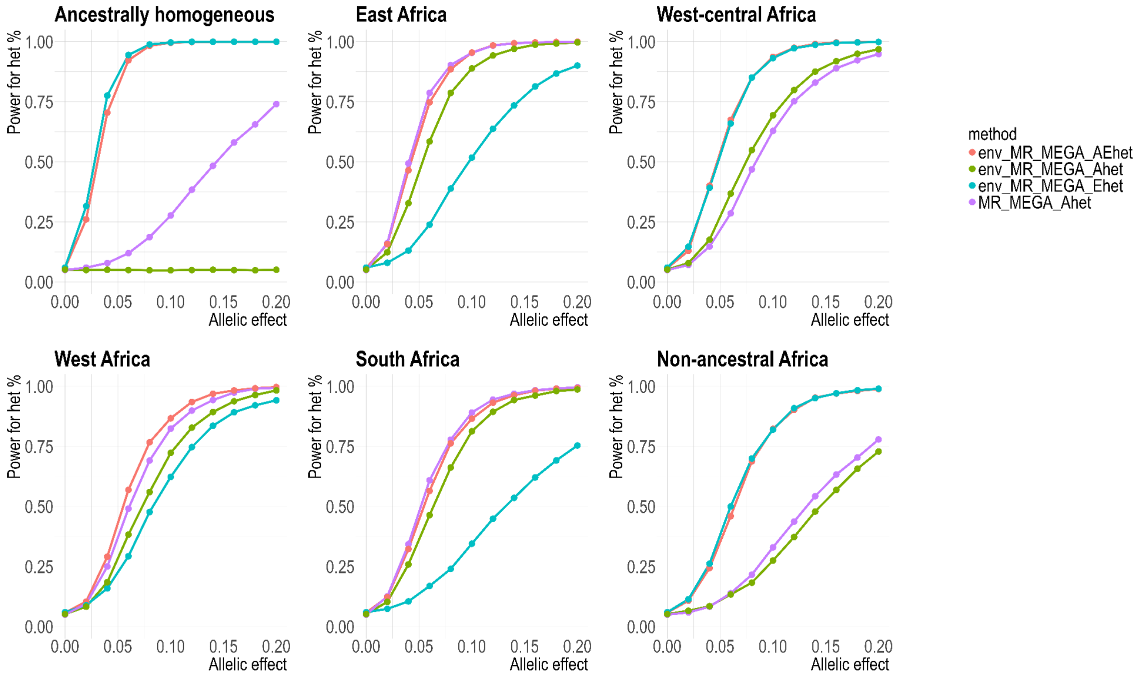

Supplementary Figure 12. In the ancestrally homogeneous scenario, power to detect association by env-MR-MEGA was higher at causal variants than at their tag variants. Tag variants had similar LD with the causal variants in populations from the same region but differed between regions. The power to detect allelic heterogeneity due to ancestry and environment or due to environment alone was notably greater at causal variants than at their tag SNPs. The largest difference between tests at causal variants and tag SNPs is in the power for allelic heterogeneity due to ancestry alone. At causal variants, the nominal significance level is achieved for allelic heterogeneity due to ancestry alone, while this power is notably higher at tag SNPs. Data are presented as the proportion of replications in which the causal variant is genome-wide significant ($P < 5 \times 10^{-8}$) ± SEM, where SEM is the standard proportion error bound of a 95% confidence interval based on 1000 replications.

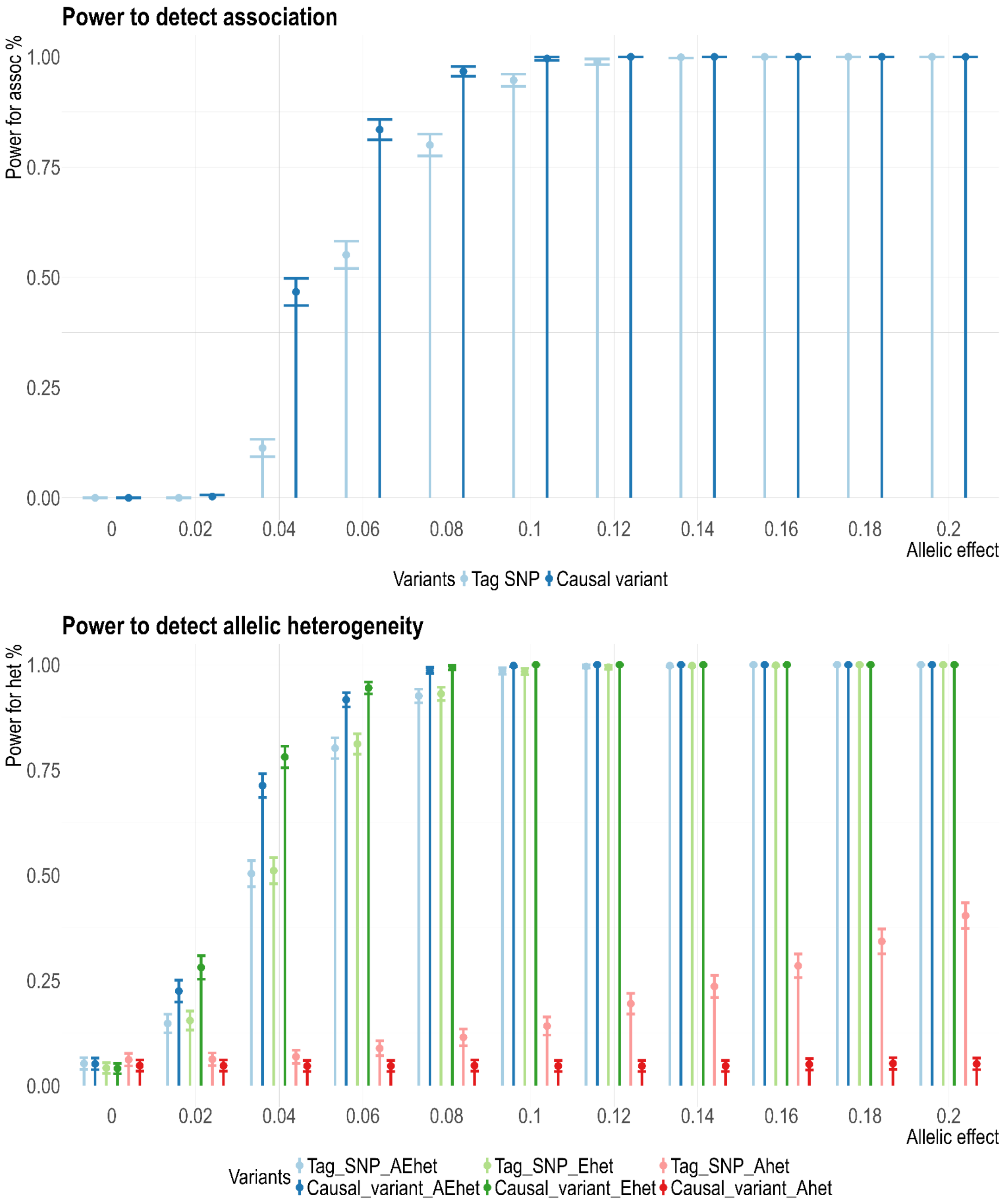

Supplement: Supplementary file 2 — Supplemental Information [file 42003_2024_7236_MOESM2_ESM.pdf]
