# Supplementary material for: Accounting for heterogeneity due to environmental sources in meta-analysis of genome-wide association studies
Source: Commun Biol. 2024 Nov 14;7:1512. doi: 10.1038/s42003-024-07236-9 (PMC11564974; doi:10.1038/s42003-024-07236-9)
Supplement: Supplementary file 3 — Description of Additional Supplementary Files [file 42003_2024_7236_MOESM3_ESM.pdf]

Description of Additional Supplementary Files

File name: Supplementary Data 1.1

Description: Overview of cohorts

File name: Supplementary Data 1.2

Description: Genetic Variants in twelve sex-stratified African cohorts overlapping with African/American Admixed Ancestries in GLGC Dataset: MR-MEGA and env-MR-MEGA Analyses for LDL-Cholesterol

File name: Supplementary Data 1.3

Description: Illustration of SNPs showing heterogeneity due to sex (rs12740374) and urban status (rs373518)
